# Development of a highly efficient Axiom™ 70 K SNP array for *Pyrus* and evaluation for high-density mapping and germplasm characterization

**DOI:** 10.1186/s12864-019-5712-3

**Published:** 2019-05-02

**Authors:** Sara Montanari, Luca Bianco, Brian J. Allen, Pedro J. Martínez-García, Nahla V. Bassil, Joseph Postman, Mareike Knäbel, Biff Kitson, Cecilia H. Deng, David Chagné, Marc W. Crepeau, Charles H. Langley, Kate Evans, Amit Dhingra, Michela Troggio, David B. Neale

**Affiliations:** 1 Department of Plant Sciences, University of California, Davis, CA USA; 2 Research and Innovation Centre, Fondazione Edmund Mach, San Michele all’Adige, Trento, Italy; 3 USDA Agricultural Research Service, National Clonal Germplasm Repository, Corvallis, OR USA; 4 Palmerston North Research Centre, The New Zealand Institute for Plant & Food Research Limited (PFR), Palmerston North, New Zealand; 5 Motueka Research Centre, The New Zealand Institute for Plant & Food Research Limited (PFR), Motueka, New Zealand; 6 Auckland Research Centre, The New Zealand Institute for Plant & Food Research Limited (PFR), Auckland, New Zealand; 7 Department of Evolution and Ecology, University of California, Davis, CA USA; 8 Tree Fruit Research and Extension Center, Washington State University, Wenatchee, WA USA; 9 Department of Horticulture, Washington State University, Pullman, WA USA

**Keywords:** *Pyrus*, Single nucleotide polymorphism, Genotyping, Germplasm, Genetic diversity, Breeding

## Abstract

**Background:**

Both a source of diversity and the development of genomic tools, such as reference genomes and molecular markers, are equally important to enable faster progress in plant breeding. Pear (*Pyrus* spp.) lags far behind other fruit and nut crops in terms of employment of available genetic resources for new cultivar development. To address this gap, we designed a high-density, high-efficiency and robust single nucleotide polymorphism (SNP) array for pear, with the main objectives of conducting genetic diversity and genome-wide association studies.

**Results:**

By applying a two-step design process, which consisted of the construction of a first ‘draft’ array for the screening of a small subset of samples, we were able to identify the most robust and informative SNPs to include in the Applied Biosystems™ Axiom™ Pear 70 K Genotyping Array, currently the densest SNP array for pear. Preliminary evaluation of this 70 K array in 1416 diverse pear accessions from the USDA National Clonal Germplasm Repository (NCGR) in Corvallis, OR identified 66,616 SNPs (93% of all the tiled SNPs) as high quality and polymorphic (*PolyHighResolution*). We further used the Axiom Pear 70 K Genotyping Array to construct high-density linkage maps in a bi-parental population, and to make a direct comparison with available genotyping-by-sequencing (GBS) data, which suggested that the SNP array is a more robust method of screening for SNPs than restriction enzyme reduced representation sequence-based genotyping.

**Conclusions:**

The Axiom Pear 70 K Genotyping Array, with its high efficiency in a widely diverse panel of *Pyrus* species and cultivars, represents a valuable resource for a multitude of molecular studies in pear. The characterization of the USDA-NCGR collection with this array will provide important information for pear geneticists and breeders, as well as for the optimization of conservation strategies for *Pyrus*.

**Electronic supplementary material:**

The online version of this article (10.1186/s12864-019-5712-3) contains supplementary material, which is available to authorized users.

## Background

Single nucleotide polymorphisms (SNPs) and insertion/deletions (INDELs) are the most abundant classes of genetic variation in plant genomes [1]. Recent advancements in sequencing and high-throughput genotyping technologies have greatly accelerated the discovery and profiling of millions of SNPs in many species [2]. Today, SNPs are the markers of choice for linkage and quantitative trait locus (QTL) mapping, and they have enabled the dissection of important traits in species with complex and highly heterozygous genomes [3–7]. These tools have also enabled genome-wide association studies (GWAS) in outcrossing species, which require several thousands of SNPs, because of the rapid linkage disequilibrium (LD) decay [8–10], and the implementation of genomic selection (GS) in a range of crops [11–13]. Furthermore, high-throughput SNP genotyping has proven useful in the study of the genetic diversity of natural populations and germplasm collections, the elucidation of aspects of plant domestication and evolution, and has important implications for both breeding and conservation [14–18].

Pear (*Pyrus* sp.) is one of the most important fruit tree crops in temperate climate regions. Despite the existence of a number of pear breeding programs internationally (for example Lespinasse et al. [19], Musacchi et al. [20], White and Brewer [21]), and recent progress towards the implementation of genomics into breeding [22–24], pear lags far behind other temperate fruit and nut crops (such as apple and peach) in terms of available genetic resources and efficiency of new cultivar development [25, 26]. In the last few years, various technologies were applied in pear for SNP discovery and genotyping, including array development [27, 28] and different genotyping-by-sequencing (GBS) strategies [18, 29–32]. These technologies have enabled the construction of linkage maps in different families, including an integrated high-density consensus map [32], and the discovery of QTLs for control of a number of important traits [7, 33–37]. However, examples of successful application of marker-assisted selection (MAS) in pear are still lacking, and GWAS and GS are limited by the very low number of markers available. As demonstrated by Kumar et al. [31] and by Wu et al. [18] the LD decay is very rapid in *Pyrus.* In the presence of rapid LD decay, the power of GWAS and the accuracy of GS increase with higher marker densities and larger samples sizes [11, 38, 39]. New tools that enable high-density and large-scale genotyping are essential to ensure faster progress in pear breeding.

The USDA National Clonal Germplasm Repository (NCGR) in Corvallis, OR, maintains 2300 clonal pear accessions and 364 seed lots with origins in 55 countries, representing nearly every known *Pyrus* species (GRIN, 07-16-2018: https://npgsweb.ars-grin.gov/gringlobal/search.aspx). This collection provides a valuable source of diversity for exploitation in pear breeding programs. Subsets of this collection have been genotyped with microsatellite markers, enabling synonym identification, elucidation of relationship patterns [40–42], and description of the extent of *P. communis* genetic diversity [43]. Nevertheless, a thorough genetic characterization of this germplasm and of the variation between and within the different species held at the repository is still missing.

In this study, our objectives were to develop a genome-wide high-density *Pyrus* SNP array that is informative for both the genetic characterization of the diverse NCGR collection, as well as GWAS and QTL mapping analysis. Among the various technologies available, SNP arrays have the advantage of providing complete and reliable genotypic data without requiring a preliminary complex bioinformatics processing, as required for GBS methods [44–46]. SNP arrays at various densities have been developed for many plant species (poplar [47], pea [48], maize [46], chickpea [44], to cite some of the most recent), including several Rosaceae (e.g., apple [45, 49], rose [50], strawberry [51]). Here, we designed a high-efficiency Applied Biosystems™ Axiom™ Pear 70 K Genotyping Array and validated it by genotyping almost the entire NCGR collection and two F1 pear populations.

## Results

### Read alignment and SNP calling by group of species

**Fig. 1:**
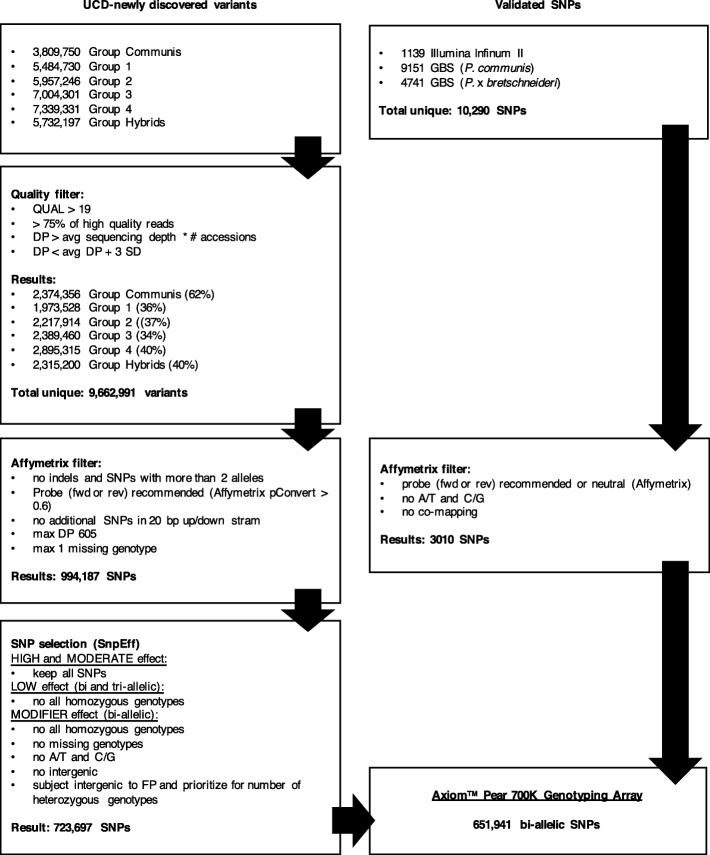
Schematic representation of the filters applied to design the Axiom™ Pear 700 K Genotyping Array. The diagram on the left-hand side shows the different steps of the Quality filter, the Affymetrix filter and the SNP selection applied through the pathway from the initial number of new variants discovered to the final set of SNPs tiled on the Axiom Pear 700 K Genotyping Array. The diagram on the right-hand side shows the Affymetrix filtering steps applied to the validated SNPs prior to inclusion in the Axiom Pear 700 K Genotyping Array. The total number of variants at each step are reported

Sequencing of 55 pear accessions, representing cultivars, founders and wild species, for a total of 29 different *Pyrus* species and interspecific hybrids (Additional file 1), resulted in an average sequencing coverage of 5.0x per sample, ranging from 3.8x (CPYR 828.001) to 6.0x (US 309), after quality and adapter-trimming. Read mapping, SNP calling and the application of the Quality filter yielded different numbers of variants for each of the six groups of species we had identified (Fig. 1). In total, a unique set of 9.7 M variants passed the Quality filter, and they were further reduced to approximately 1 M bi-allelic SNPs (10%) after the Affymetrix filter (Fig. 1). We also applied the Affymetrix filter to a set of 10,290 validated SNPs (from the apple and pear Illumina Infinium® II 9 K SNP array [27] and from GBS data developed at The New Zealand Institute for Plant & Food Research Limited (PFR) [32]), which were then reduced to 3010 (29%).

### Selection of 700 K SNPs for the first “draft” axiom Array and assessment of their performance

**Fig. 2:**
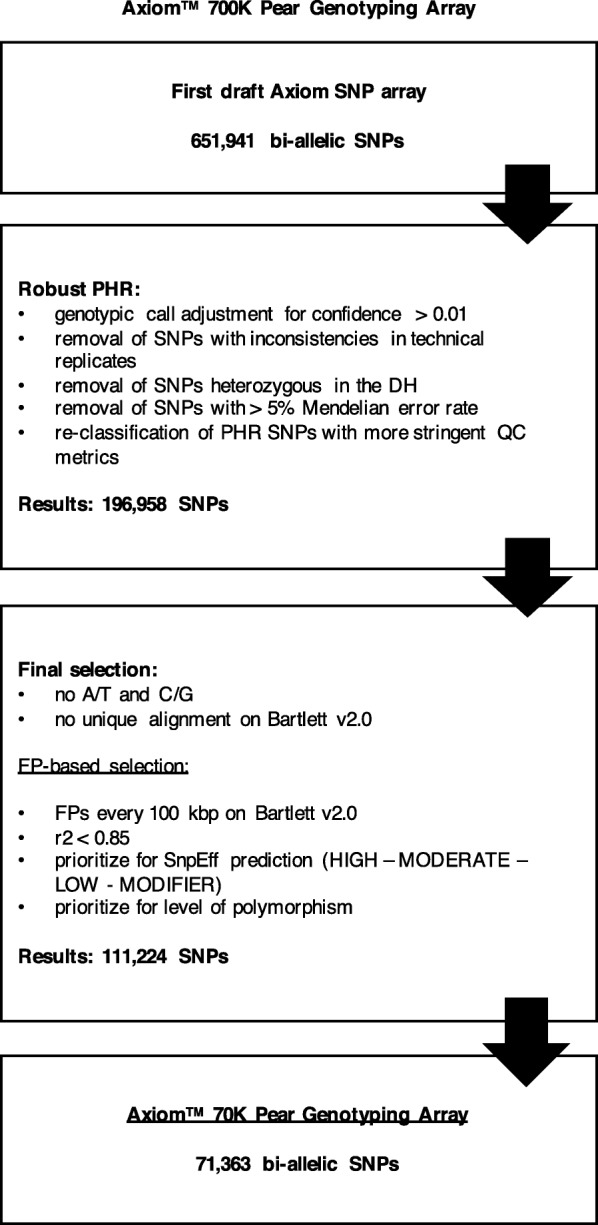
Schematic representation of the filters applied to design the Axiom™ Pear 70 K Genotyping Array. This diagram shows the different steps applied for the identification of the robust *PolyHighResolution* (PHR) SNPs on the Axiom Pear 700 K Genotyping Array, and the subsequent selection of the highly-informative SNPs tiled on the Axiom Pear 70 K Genotyping Array. The total numbers of variants at each step are reported

We successfully annotated 989,566 newly discovered SNPs using the ‘Bartlett’ v1.0 gene predictions and the software SnpEff [52]. Among these, 84,509 were non-synonymous mutations with HIGH and MODERATE impact, and 100,191 SNPs were classified as LOW impact. HIGH impact variants cause changes in start or stop codons, or hit splice sites, therefore they have a disruptive impact in the protein, MODERATE are non-disruptive mutations that might change protein effectiveness, while LOW impact variants are synonymous mutations or non-synonymous that are assumed to be mostly harmless (Additional file 2). The HIGH and MODERATE impact SNPs were prioritized on the array and, in addition to 93% of the LOW impact SNPs, they were successfully tiled (Table 1). The majority of the SNPs were classified as MODIFIER, which are located between genes or in non-coding regions of a gene (Additional file 2). A total of 471,625 of them were incorporated in the array: 447,790 were SNPs located downstream or upstream of a gene (within 5 Kbp of it) or in intragenic or intronic regions, while 23,835 SNPs were intergenic (at > 5 Kbp of distance). Using a Focal Point (FP) strategy as in Chagné et al. [53], intergenic SNPs were chosen to be widely distributed across the ‘Bartlett’ v1.0 genome. We also submitted all the 3010 validated SNPs from previous studies, except for 44 GBS SNPs that were excluded for technical reasons. In total, we submitted 726,707 SNPs to Affymetrix (now part of Thermo Fisher Scientific), and 651,941 were successfully tiled on the Axiom 700 K Pear Genotyping Array. Following a strategy similar to that of Unterseer et al. [46], this array was not built with the intention of commercialization, but as a first “draft” array for the identification of the highest quality, most informative SNPs. To fulfill this objective, we selected 284 diverse pear accessions (plus four technical replicates) to constitute the screening panel (Additional file 3) for genotyping with the Axiom 700 K Pear Genotyping Array. Approximately 5% of the samples (13) failed to pass the 97% Quality Control Call Rate (QC CR) threshold, and were excluded from genotyping. A total of 391,892 SNPs (60% of total tiled SNPs) were classified as *PolyHighResolution* (PHR), according to the Affymetrix default parameters for diploid samples: these are highly polymorphic SNPs exhibiting all three genotypic classes with a good cluster resolution. After adjusting the genotypic calls with a more stringent confidence threshold, the number of PHR SNPs was reduced to 315,642 (Table 2). We next removed 384 SNPs because of inconsistencies across the technical replicates, or because they were called heterozygous in the double haploid (DH) of ‘Bartlett’ (a.k.a. ‘Williams’ Bon Chrétien’). Investigation of possible Mendelian errors in 22 trios (comprised of the two parents, P01 and P02, and one offspring, Off) (Additional file 4), resulted in the elimination of two false trios (OHxLBJ and IxY) and the subsequent identification of 14,189 SNPs with an error rate higher than 5%. Final filtration of the remaining 301,069 PHR SNPs with more stringent metrics left 196,958 SNPs, which we define in this manuscript as robust PHR. They represent 30% of the initial number of SNPs tiled on the Axiom Pear 700 K Genotyping Array (Fig. 2).

**Table 1:** Number of SNPs for different classes reported by SnpEff

	#SNPs annotated with SnpEff	#SNPs submitted	#SNPs tiled on the 700 K array	#SNPs classified as robust PHR	#SNPs tiled on the 70 K array
UCD-newly discovered SNPs	989,566	723,697	648,975	196,640	71,182
HIGH^a^	1746	1746	1746	415	221
MODERATE^b^	82,763	82,763	82,763	31,559	14,471
LOW^c^	100,191	92,841	92,841	41,526	22,750
MODIFIER^d^ non-intergenic	583,645	447,790	447,790	120,839	33,647
MODIFIER^d^ intergenic	221,221	98,557	23,835	2301	93
Validated SNPs	–	3010	2966	318	181
Infinium® II 9 K SNP array	–	558	558	220	122
GBS *P. communis*	–	1440	1440	80	49
GBS *P.* × *bretschneideri*	–	1012	968	18	10
Totals	–	726,707	651,941	196,958	71,363

^a^ HIGH = The variant is assumed to have high (disruptive) impact in the protein, probably causing protein truncation, loss of function or triggering nonsense-mediated decay

^b^ MODERATE = A non-disruptive variant that might change protein effectiveness

^c^ LOW = Assumed to be mostly harmless or unlikely to change protein behavior

^d^ MODIFIER = Usually non-coding variants or variants affecting non-coding genes, where predictions are difficult or there is no evidence of impact

The newly discovered SNPs that passed the Affymetrix filter were annotated with the software SnpEff, which reported their predicted impact on the protein (HIGH, MODERATE, LOW or MODIFIER). The number of SNPs for each class is shown for all annotated SNPs, the final set of SNPs submitted to Affymetrix to build the first “draft” array, the SNPs tiled on the Axiom™ Pear 700 K Genotyping Array, the robust *PolyHighResolution* (PHR) SNPs of this array, the SNPs tiled on the Axiom Pear 70 K Genotyping Array. The numbers of validated SNPs are also shown

**Table 2:** Number of SNPs for different Axiom™ SNP categories

SNP category	700 K array	70 K array
*PolyHighResolution* (PHR)	315,642 (48.4%)	66,616 (93.3%)
*CallRateBelowThreshold* (CRBT)	80,257 (12.3%)	114 (0.2%)
*HomHomResolution* (HHR)	2 (0%)	0 (0%)
*MonoHighResolution* (MHR)	4075 (0.6%)	68 (0.1%)
*NoMinorHom* (NMH)	32,634 (5%)	663 (0.9%)
*Other*	137,748 (21.1%)	191 (0.3%)
*OffTargetVariant* (OTV)	51,981 (8%)	78 (0.1%)
*AAvarianceX*	3695 (0.6%)	537 (0.8%)
*AAvarianceY*	3884 (0.6%)	538 (0.8%)
*ABvarianceX*	3613 (0.6%)	653 (0.9%)
*ABvarianceY*	8566 (1.3%)	533 (0.7%)
*BBvarianceX*	4773 (0.7%)	821 (1.1%)
*BBvarianceY*	5071 (0.8%)	551 (0.8%)
Total	651,941	71,363

SNPs from the Axiom arrays are classified into 13 categories, depending on their metrics. The number of SNPs for each category are shown for the Axiom Pear 700 K Genotyping Array (after call adjustment with a confidence threshold of 0.01) and for the Axiom Pear 70 K Genotyping Array

### Selection of highly informative SNPs for the axiom 70 K pear genotyping Array

After BLASTing the 70 bp-region flanking each of the tiled 651,941 SNPs to the current (May 2017) version of the ‘Bartlett’ v2.0 genome (G. Linsmith, unpublished), 580,621 exhibited a unique alignment, covering 208 (out of ~ 1400) contigs and 470 (out of ~ 500) Mbp. Among those, 181,022 were robust PHR (after exclusion of 7690 A/T and C/G SNPs). Division of these 181,022 SNPs into eight classes of polymorphism based on their minor allele frequency (MAF) values, as reported in Table 3, resulted in 24,518 SNPs highly polymorphic (0.2 ≥ MAF ≤ 0.8) across the entire screening panel (classes “HighlyPoly”, “Poly_Discr” and “Poly”), and 57,635 within the Group Communis (classes “HighlyPoly”, “PolyComm_Discr” and “PloyComm”). We also identified 29,758 SNPs with different minor alleles in groups with MAF 0.2 and MAF 0.8 (classes “PolyComm_Discr”, “Poly_Discr” and “LowPoly_Discr”), 25,797 of which could discriminate between European (Communis, Group 1 and Group 2) and Asian species (Groups 3 and 4). In general, we identified SNPs that were able to discriminate between each pair of groups, except for Communis and Group 1 (Table 4). In addition, 10 robust PHR SNPs that were associated with important agronomic traits [7, 35] were given high priority, and four more were selected from other SNP categories, after visual evaluation of their cluster plots.

**Table 3:** Classes of polymorphism established for the robust *PolyHighResolution* (PHR) SNPs

Priority	Class	Explanation	# tot SNPs	# tiled SNPs
1	Traits	SNPs associated with important agronomic traits	10 (0.0%)	14 (0%)
2	HighlyPoly	Highly polymorphic across the entire screening panel and within each Group	2499 (1.4%)	2232 (3.1%)
3	PolyComm_Discr	Highly polymorphic within the Group Communis and able to discriminate among two or more other Groups	3025 (1.7%)	2153 (3%)
4	PolyComm	Highly polymorphic within the Group Communis	52,111 (28.8%)	27,936 (39.1%)
5	Poly_Discr	Highly polymorphic across the entire screening panel and within one or more Groups, excluding Communis, and able to discriminate among two or more other Groups	19,459 (10.7%)	6063 (8.5%)
6	Poly	Highly polymorphic across the entire screening panel and within one or more Groups, excluding Communis	2560 (1.4%)	2220 (3.1%)
7	LowPoly_Discr	Highly polymorphic within one or more Groups, excluding Communis, and able to discriminate among two or more other Groups	7274 (4%)	3381 (4.7%)
8	LowPoly	Highly polymorphic only within one or two Groups, excluding Communis	94,084 (52%)	27,364 (38.3%)
Total			181,022	71,363

Robust PHR SNPs from the Axiom™ Pear 700 K Genotyping Array that had a unique alignment on the ‘Bartlett’ v2.0 genome were divided into eight classes of polymorphism. SNPs were considered highly polymorphic, across the entire screening panel or within a group of species, when they had minor allele frequency (MAF) values of 0.2 ≤ MAF ≤ 0.8. SNPs with different minor alleles in groups of species with MAF 0.2 and MAF 0.8 were considered discriminative. SNPs of class Traits are SNPs from the Infinium® II chip that had been associated with important agronomic traits. This Table presents the acronym for each class, ordered by priority, and their respective descriptions, as well as the number of total SNPs in each class and the numbers finally tiled on the Axiom Pear 70 K Genotyping Array

**Table 4:** Numbers of SNPs able to discriminate between each pair of groups of species

Robust PHR	Communis	Group 1	Group 2	Group 3	Group 4
Communis					
Group 1	0				
Group 2	1022	626			
Group 3	22,929	21,571	19,111		
Group 4	18,833	17,466	15,116	2711	
Tiled SNPs	Communis	Group 1	Group 2	Group 3	Group 4
Communis					
Group 1	0				
Group 2	452	248			
Group 3	7953	6979	6292		
Group 4	5455	4488	3860	1079	

SNPs with different minor alleles in groups of species with minor allele frequency (MAF) values of MAF 0.2 and MAF 0.8 were considered discriminative and grouped into the classes PolyComm_Discr, Poly_Discr or LowPoly_Discr. We combined the information from these classes to compute the numbers of SNPs able to discriminate between each pair of groups of species. This is shown for both the robust *PolyHighResolution* SNPs from the Axiom™ Pear 700 K Genotyping Array that had a unique alignment on the ‘Bartlett’ v2.0 genome, and the SNPs tiled on the Axiom Pear 70 K Genotyping Array. Multiple entries per SNP are possible

A total of 111,224 SNPs was left after removal of those in high LD. This list of SNPs was sorted in order of priority, based on their distribution across the scaffolds of the ‘Bartlett’ v2.0 genome (using again the FP strategy), as well as their SnpEff prediction and classification into degrees of polymorphism, and was submitted to Affymetrix (Fig. 2). A total of 71,363 SNPs was successfully tiled on the Axiom Pear 70 K Genotyping Array. The distribution of these SNPs on the chromosomes of *P. communis* was evaluated using the anchored portion of the first version of the ‘Bartlett’ genome [32, 54], since at the current stage a physical map for ‘Bartlett’ v2.0 has not yet been developed. All SNPs were aligned to the genome, and only two were eliminated after filtering. A total of 69,187 SNPs had unique alignment, of which 56,479 were located on one of the 17 chromosomes and 12,708 fell on unanchored scaffolds (Additional file 5). The distribution across the anchored portion of the *P.* x *bretschneideri* ‘Dangshansuli’ genome [55] was evaluated as well. While just 17 SNPs did not align to this genome, 13,528 more were eliminated at filtering, and only 43,816 had unique alignment. Of these, 41,179 fell on chromosomes and 2637 on unanchored scaffolds (Additional file 5). Between 1858 and 6127 and between 1382 and 5267 SNPs were located on each chromosome of ‘Bartlett’ and *P. bretschneideri*, respectively; in both genomes, chromosome 1 was the one with less SNPs, and chromosome 15 the one with the most. Chromosome 15 is also the longest one in both genomes.

**Fig. 3:**
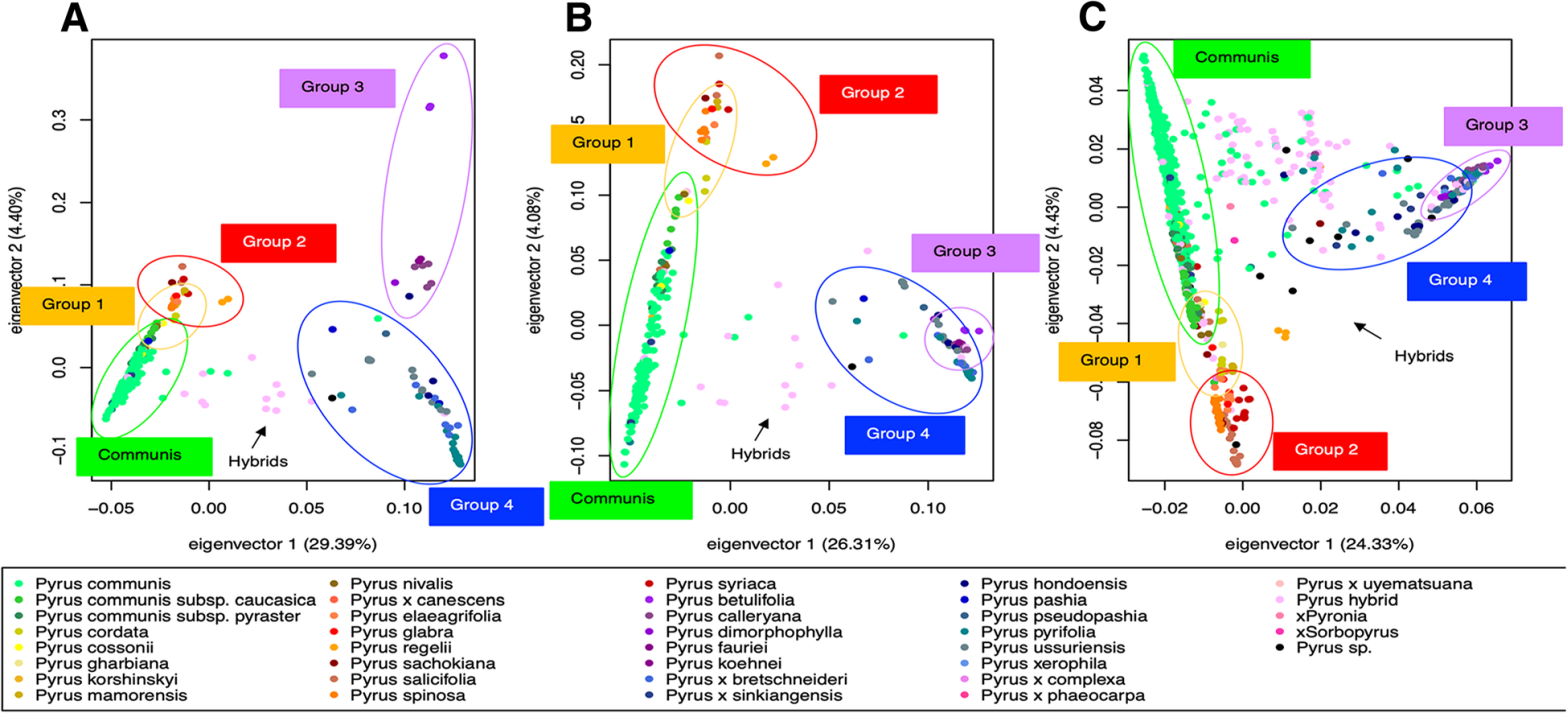
Principal Component Analysis (PCA) plots. The PC1 versus PC2 plots are reported. **a** PCA performed with all robust *PolyHighResolution* (PHR) SNPs for the samples of the screening panel. **b** PCA performed with the SNPs tiled on the Axiom™ Pear 70 K Genotyping Array for the samples of the screening panel. **c** PCA performed with the PHR SNPs of the Axiom Pear 70 K Genotyping Array for all genotyped pear accessions, including both the screening and the genotyping panel. A different color is used for each *Pyrus s*pecies, and the clusters of each group of species are highlighted. Group Communis = *P. communis*; Group 1 = *P. communis* wild relatives; Group 2 = Middle East/Central Asia arid-adapted species; Group 3 = East Asian “pea” pears; Group 4 = East Asian large-fruited cultivars and wild relatives; Group Hybrids = interspecific hybrids

Evaluation of the population structure of the screening panel using first all robust PHR SNPs, and then the subset of highly-informative SNPs that were to be included in the Axiom Pear 70 K Genotyping Array, verified the potential of the selected markers to depict germplasm diversity. After running the Concordance Check on 275 passing samples of the screening panel, we identified 19 groups of duplicates (Additional file 6). In total, 255 samples were unique and were used for Principal Component Analysis (PCA) with both the 196,958 robust PHR SNPs and the 71,363 SNPs tiled on the 70 K array. PC1 accounted for 29.39% of the variability with the robust PHR, and 26.31% with the selected 70 K SNPs, while the other PCs accounted for less than 4.5% of the variability with both sets of SNPs. Figure 3 shows the PC1 versus PC2 plots drawn for the robust PHR (A) and the selected SNPs (B): in both cases, the six groups of species were differentiated, with partial overlapping between Group Communis and Group 1, between Group 1 and Group 2, and between Group 3 and Group 4. Clustering of the three European groups and of Group 4 did not appear to change from one set of SNPs to the other; Group 3 samples, in contrast, were projected within the Group 4 cluster in the PC1 versus PC2 plot for the 71,363 SNPs, while the two groups were well separated when using all robust PHR SNPs. However, Groups 3 and 4 could still be clearly differentiated on examination of PC1 versus PC3 and PC2 versus PC3 plots (Additional file 7A and Additional file 7B). Furthermore, three *P. betulaefolia* samples that were outliers from Group 3 showed similar behavior in both PCAs (Fig. 3a and b). The interspecific hybrids (Group Hybrids) were mostly located in between the European (Groups Communis, 1 and 2) and the Asian (Groups 3 and 4) pear samples.

### Validation of the axiom 70 K pear genotyping Array by large-scale genotyping of a diverse Pyrus germplasm collection

A total of 141 samples out of the 1416 included in the genotyping panel (10%) failed at sample QC when screened with the Axiom Pear 70 K Genotyping Array and were excluded from genotyping (Additional file 8). The genotypic calls for the remaining samples were generated using the Affymetrix default parameters for diploid species, resulting in 66,616 SNPs being classified as PHR (93% of total tiled SNPs) (Table 2). After visual inspection of cluster plots for 1000 random PHR SNPs, we decided that call adjustment and more stringent filtering were not necessary, since all appeared robust. The reproducibility rate for these PHR SNPs was very high, averaging 99.9% for all technical and biological replicates included in the panel. Furthermore, some samples from the screening panel that were genotyped with the 700 K array were also re-genotyped with the 70 K array and the calls were 99.9% concordant (on average).

Analysis of the population structure of all genotyped samples (1177 after merging of the screening and genotyping panels, and removal of duplicates) gave rise to similar results to the PCA run just on the screening panel with the 71,363 tiled SNPs (Fig. 3b and c): all groups of species could be clearly differentiated in the PC1 versus PC2 plot, except for Groups 3 and 4, which separated well in the PC1 versus PC3 and PC2 versus PC3 plots (Additional file 7C). In all three PC1 versus PC2 plots (Fig. 3) samples that appeared to cluster within the wrong group of species could be clearly spotted.

### High-density linkage maps and comparison of the axiom 70 K pear genotyping Array with GBS data

When we used the 70 K SNP array to genotype two F1 interspecific families developed at PFR from *P. communis*, *P.* x *bretschneideri* and *P. pyrifolia* accessions, P16.009 and P493, a total of 29,935 markers were polymorphic in the first population, which comprised 19,863, 4360 and 4864 SNPs that were informative for either the female, the male or both parents, respectively. Parental maps were constructed using SNP markers segregating in a backcross manner, after removing identical markers that co-segregated. In total, 1209 and 1010 unique markers mapped in P16.009_female and P16.009_male, respectively. Linkage maps were calculated and spanned 1236.9 and 1444.1 cM, with an average marker distance of 1.13 and 1.58 cM, respectively (Additional file 9 and Additional file 10). The remaining unmapped markers were not linked to any group.

**Fig. 4:**
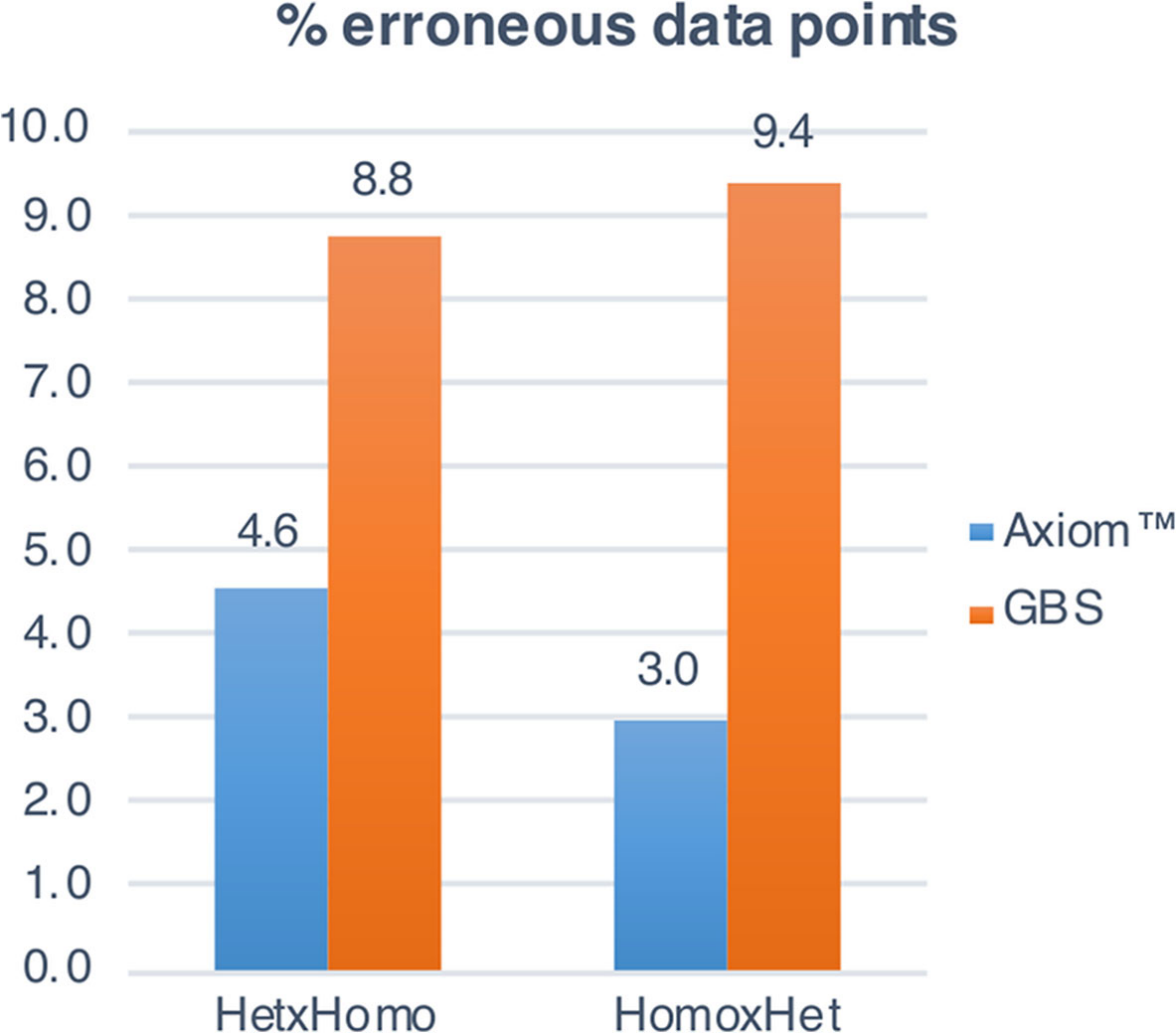
Comparison of SNP array and GBS (genotyping-by-sequencing) error rate. The percentage of erroneous data points for heterozygous × homozygous (Het × Homo) and homozygous × heterozygous (Homo × Het) SNP markers in the P493 population are plotted. The error rate for the Axiom™ 70 K Pear Genotyping Array data is depicted in blue, and the GBS data error rate in orange

We could not construct the parental maps of P493 because this population had only 16 offspring. However, availability of restriction enzyme-based GBS [56] data for these individuals allowed us to make a direct comparison between array and GBS SNPs. In total, 25,147 Axiom markers segregated in this population, but only 16,369 GBS SNPs. Furthermore, 19,424 (9.1%) genotypic data points were suspicious and probably erroneous for the GBS SNPs, while the error rate was significantly lower (Kruskal Wallis test, *ρvalue* = 0.0016) for the Axiom data set (13,750 data points, corresponding to 3.7%). These observations were consistent among segregating types (Fig. 4 and Additional file 11).

### Evaluation of additional classes of SNPs

While the classes *CallRateBelowThreshold* and *Other* identify low quality SNPs, and the class *MonoHighResolution* the monomorphic SNPs, all the remaining SNP categories include polymorphic and potentially useful markers. From the large-scale genotyping with the 70 K array, only 4374 SNPs (6% of total SNPs) fell into one of these classes of polymorphism (Table 2), but the majority of them showed poor cluster resolution. The proportion of these poorly resolved SNPs was higher when we genotyped the screening panel with the 700 K array, for which 114,217 SNPs (18% of total SNPs) were classified into one of these categories. When we visually evaluated the cluster plots of 1000 random SNPs from each category, we concluded that only the *NoMinorHom* (NMH), *ABvarianceY* and *OffTargetVariants* were worth undergoing additional analysis, while the majority of the SNPs in the other classes had poor-quality clusters. NMH SNPs usually have good cluster resolution, but one of the two homozygous clusters is missing. A total of 32,634 SNPs was classified as NMH. With the objective of eliminating any possible genotyping error, we filtered these SNPs with stringent metric thresholds, as used previously for the robust PHR, and we checked their Mendelian error rate in the 20 true trios. A total of 23,124 SNPs passed the stringent filter (71% of total NMH), and only 30 of them had more than 5% Mendelian errors. The *ABvarianceY* SNPs (with high heterozygous cluster variance in the *y* dimension) are similar to the *OffTargetVariance* SNPs, which display an additional cluster at a low hybridization intensity just below the AB cluster (Additional file 12). These SNPs tag sites whose sequence is significantly different from the marker probe for a number of samples, which therefore group in an additional cluster. When we ran the *OTV_Caller* in “SNPolisher” on the *OffTargetVariance* (51,981) and *ABvarianceY* (8566) SNPs (which we refer to as OTV), we generated new genotypic calls for the fourth, additional cluster (samples are coded as − 2). On using a similar approach as for the NMH SNPs, a total of 15,719 SNPs passed the stringent filter (26% of the 60,547 OTV), 2113 of which had a Mendelian error rate higher than 5%. We finally displayed the cluster plots for the 13,606 good OTV SNPs, using different colors for each species, and we observed that Asian *Pyrus* species were more likely to fall into the fourth OTV cluster (Additional file 13).

## Discussion

### Development of the most efficient high-resolution SNP array for fruit tree crops

The Axiom Pear 70 K Genotyping Array is currently the largest SNP array for pear, and one of the densest for fruit tree crops, second only to the Axiom Apple480K Array [45]. It is also the most efficient SNP array for tree crops, with a conversion rate of 93%, thanks to the two-step design we performed. Unterseer et al. [46] applied the same strategy when developing the Axiom Maize Genotyping Array, whose 616,201 variants were chosen from a set of 1.2 M variants by screening a broad genetic diversity panel, and they observed a proportion of PHR SNPs (92%) similar to that found in the present study. Our decision to apply this particular design strategy was driven by a number of technical limitations and challenges that we faced at the beginning of the study: i) the low sequencing coverage of the discovery panel; ii) the broad genetic diversity of the discovery panel, which included 29 different *Pyrus* species and hybrids, selected with the objective of designing an array that could be used to genotype the entire *Pyrus* NCGR collection; iii) the duplicated nature of the *Pyrus* genome; and iv) the availability, at the beginning of our study, of a draft, fragmented reference genome constructed from the highly heterozygous ‘Bartlett’. Because of all these issues, we anticipated a high probability of error both at the read-alignment and the SNP calling phases, which could have been only partially controlled with the Quality filter. In contrast, SNPs selected from the initial set based on their performance in genotyping a screening panel would be expected to be more reliable. The low proportion of robust PHR SNPs from the 700 K array (30%), and the subsequent very high proportion from the final 70 K array (93%) eventually confirmed our hypotheses. In comparison, the Axiom Apple480K Array, which was designed from the high-depth re-sequencing of 63 *Malus* x *domestica* cultivars, had a proportion of 74% PHR SNPs, 54% of which were further classified as very robust and validated PHR SNPs [45]. Although this percentage corresponds to 261,972 SNPs, a number much higher than the 66,616 SNPs in this 70 K pear array, our design makes the repeated use of the Axiom Pear 70 K Genotyping Array very cost efficient. Moreover, we observed that all the PHR SNPs of the 70 K array were also robust, making the analysis of the genotypic data more straightforward and reproducible across different studies.

Approximately 10% of the samples screened with the 70 K array failed at genotyping, mostly at the QC CR step. According to the Axiom genotyping design, samples are clustered twice and those with < 97% QC call rate during the initial round of clustering are eliminated. While often sample failure can be attributed to bad quality DNA, low CR at clustering can also occur because of the high divergence from the reference genome used to design the probes. This possibility is discussed more in detail later on. With caution, the QC CR default threshold could be lowered in cases of wide diversity germplasm.

### A genetic tool for multiple downstream analysis

The objective of this study was to design a high-density and highly efficient SNP array that could serve for multiple downstream applications, *in primis* the characterization of the large and diverse *Pyrus* germplasm and the performance of GWAS within the USDA-NCGR collection, but also for QTL mapping in bi-parental populations. The choice of accessions in the discovery and the screening panels was fundamental in our achievement of these objectives. When selecting the samples to re-sequence, we gave preference to the main founders of pear breeding programs, also trying to include a wide diversity of species. Even though the read-alignment and variant calling for samples that are highly divergent from the reference genome were more prone to errors, they were also necessary for discovery of polymorphic sites in species other than *P. communis*.

**Fig. 5:**
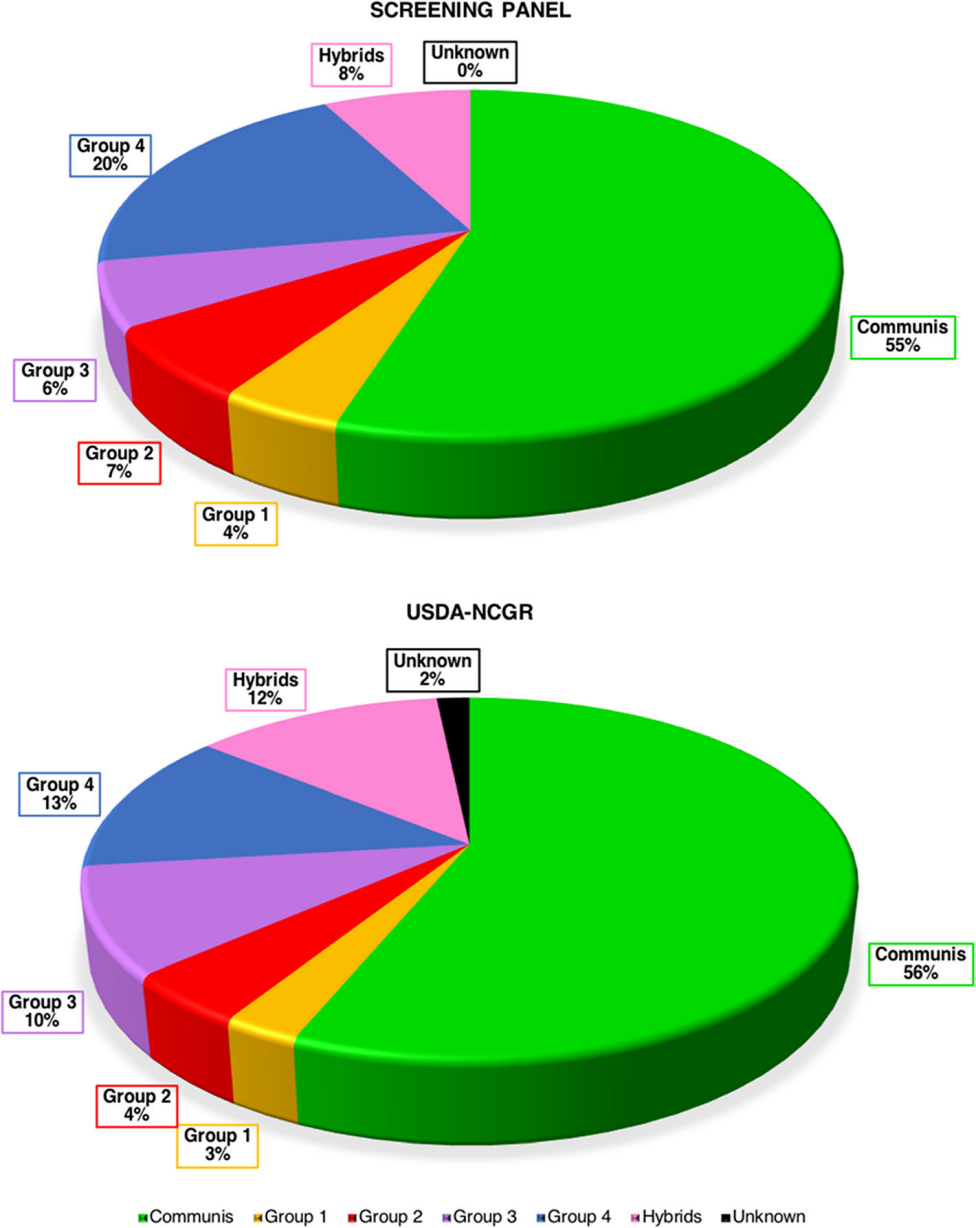
Proportions of each group of species in the screening panel and in the USDA-NCGR collection. The pie chart at the top shows the percentages of samples belonging to each group of species over the total number of samples of the screening panel. The pie chart at the bottom shows the percentages of samples belonging to each group of species over the total number of samples of the entire USDA National Clonal Germplasm Collection of Corvallis, OR. Group Communis = *P. communis*; Group 1 = *P. communis* wild relatives; Group 2 = Middle East/Central Asia arid-adapted species; Group 3 = East Asian “pea” pears; Group 4 = East Asian large-fruited cultivars and wild relatives; Group Hybrids = interspecific hybrids

The screening panel was constructed with the objective of representing as accurately as possible the relative proportions of the species held at the USDA-NCGR collection (Fig. 5 and Additional file 14). Half the accessions in this germplasm collection are *P. communis*, and our breeding programs are based mainly on crossing with *P. communis* cultivars. We therefore accepted a bias in favor of SNPs that were highly polymorphic within the Group Communis, which finally made up 45% of the 70 K array (Table 3). However, a good number of SNPs (10,515) that were highly polymorphic across the entire screening panel, and supposedly across the entire USDA-NCGR collection, was also incorporated in the 70 K array (15%). These orthologous markers, i.e. markers that are transferable across species, will enable comparative mapping among different studies and breeding programs, as well as facilitate evolutionary analysis [57]. We expect this array to be useful also for genotyping non-*P. communis* populations, since about 58% of the SNPs (41,260) were highly polymorphic within at least one of the other groups of species, as well as for the identification of genomic regions that might have been under selection, with 16% discriminative SNPs (Table 3 and Table 4). In particular, 16,771 SNPs were highly polymorphic (0.2 ≥ MAF ≤ 0.8) within the Asian cultivars (Group 4), and 18,337 within the Asian pear species in general (Groups 3 and 4), while 28,600 and 22,255 SNPs were highly polymorphic within Groups 1 and 2, respectively, representing European pear wild species.

Our goal was to identify the most informative SNPs, among the 196,958 robust PHR, for future genetic diversity studies and GWAS and QTL mapping. Hence, we selected SNPs based on their predicted effect on genes, their distribution in the genome (using a FP strategy), the non-redundancy of their genetic information (SNPs in high LD within the same FP window were removed) as well as their degree of polymorphism in the screening panel (Fig. 2). About half the newly designed SNPs incorporated in the 70 K array are in gene coding regions (Table 1), 14,692 of which (21% of the total number of SNPs) were predicted as HIGH or MODERATE impact by SnpEff. Furthermore, the 181 validated SNPs (Table 1) were all designed in coding regions [27, 32]. The proportion of coding variants in our pear array is higher than what was achieved for the Axiom Maize Genotyping Array [46], where 20% of the 600 K polymorphic sites tagged were in coding regions and only 9% (of the total) were predicted as HIGH or MODERATE effect, but lower than the 72% of the high-resolution Axiom Apple480K Array [45]. The low number of major effect-SNPs in the maize array could be a consequence of the use of inbred lines in the discovery panel, which were characterized by minimal values of heterozygosity. On the other hand, the two-step design strategy that we and Unterseer et al. [46] adopted, and that was not used in apple by Bianco et al. [45], might explain the lower proportion of coding variant’s with respect to the apple array. By carrying out a screening process, we removed all low-performing SNPs, irrespective of their SnpEff classification. Additionally, of the whole set of robust PHR SNPs on the pear 700 K array (196,958), only 37% of them were classified as HIGH, MODERATE or LOW impact (Table 1), a proportion considerably lower than the 53% observed in the final 70 K array. This outcome further supports the success of our SNP selection process. Regardless, while variants in coding regions are more likely to affect gene functions, intronic, UTR and intergenic mutations have also been associated with phenotypic traits, both in plants and in humans, probably because of high LD with unknown causal mutations [58]. On this matter, we want to underline that the MODIFIER SNPs that we chose provided non-redundant genotypic information and filled the gaps in the genome that were not covered by the other SNPs; therefore, they may be valuable in association mapping. Finally, neutral markers are useful for population structure analysis [59].

The SNPs included in the pear 70 K array were well-distributed across the 17 chromosomes of both the *P. communis* [32, 54] and the *P.* x *bretschneideri* [55] genomes (Additional file 5). A lower number of SNP markers uniquely aligning to the Chinese pear reference genome with respect to ‘Bartlett’ was expected, since the probes were designed on ‘Bartlett’. On the other hand, a higher number of SNPs fell into unanchored scaffolds in ‘Bartlett’ than in *P. bretschneideri*; however, this is in line with the different proportions of the assemblies that are currently anchored to chromosomes (50.5 and 75.5%, respectively). While the SNP-density was variable over the length of each chromosome, we could not observe any gaps, nor relevant differences among the chromosomes.

### Validation of the axiom pear 70 K genotyping Array and preliminary characterization of the Pyrus USDA-NCGR collection

The Axiom Pear 70 K Genotyping Array displayed a high degree of polymorphism across the diverse genotyped accessions from the *Pyrus* USDA-NCGR collection. This large genotypic dataset enabled the observation of strong population structure that is consistent with the geographical-based subdivision in groups of species performed by Challice and Westwood [60] (Fig. 3 and Additional file 7). The two main groups of Occidental and Oriental pears were confirmed as highly divergent. The partial overlap observed between Group Communis and Group 1, Group 1 and Group 2, and Group 3 and Group 4 reveals higher genetic similarity between these groups. According to previous phylogenetic studies [60, 61], the North African and European species belonging to Group 1 are believed to be wild relatives of *P. communis*; some West Asian species of Group 2 appeared to be related to a number of Group 1 species; and several degrees of relationship were observed between species of Groups 3 and 4.

In a recent analysis of 113 pear accessions by skim sequencing, Wu et al. [18] also observed a clear distinction between European and Asian pears, and identified subgroups within these two main categories that are in agreement with our PCA. They also reported a more detailed distinction among the Asian species that we have classified all together within Group 4. However, the objective of our PCA was merely to demonstrate the usefulness of the Axiom Pear 70 K Genotyping Array to correctly depict the genetic diversity of the genus *Pyrus*, and additional analyses are necessary to thoroughly elucidate the population structure of the *Pyrus* USDA-NCGR collection.

Since *Pyrus* is generally self-incompatible and cross-fertile, interspecific hybridization is very common in this genus [62]. While 22 species are officially recognized as primary *Pyrus* species [63], the classification of accessions with intermediate morphologies has been historically difficult in this genus. Even though molecular data have shed new light on the phylogeny of *Pyrus* [61], a large number of unresolved interspecific hybrids exists that have not been completely characterized. In this study, interspecific hybrids are easy to identify on the PCA plots, between the Occidental and the Oriental clusters, and several others are located within each group of species (Fig. 3). The genotypic information we have developed for the germplasm collection will help to clarify the origin of these hybrid accessions, and to assign the misclassified samples to the correct species.

Additionally, we were able to develop high-density and robust genetic maps for a bi-parental population, making the Axiom Pear 70 K Genotyping Array very useful for QTL mapping. The array is currently being used to genotype five more F1 populations within our breeding programs.

### SNP array data appear more robust than GBS

Many argue that SNP arrays introduce an ascertainment bias in population genetic studies whose degree depends on the diversity of the SNP discovery panel with respect to the genotyped sample set. Array-based SNPs usually penalize rare alleles, which are, instead, more easily captured using GBS [64–66]. However, in the present study we have demonstrated that such bias can be largely reduced by performing a validation of the SNPs in an array and by carefully choosing the discovery and screening panels. On the other hand, GBS is known to generate large amounts of missing data, rendering the application of genotype imputation algorithms necessary. Missing data can be reduced by increasing the sequencing coverage and the purity and quality of the DNA [67]. However, this would make GBS less suited to large-scale genotyping experiments, especially in tree crops, which are characterized by highly-heterozygous genomes and recalcitrant DNA extraction. Moreover, in the segregating population P493 we observed a larger number of polymorphic markers with the Axiom Pear 70 K Genotyping Array than with restriction enzyme-based GBS, and a significantly lower error rate, further supporting the robustness of our array design.

### Additional SNP categories may tag genomic regions of evolutionary interest

SNPs other than the high quality, highly polymorphic SNPs classified as PHR should not be discarded, in particular NMH and OTV SNPs. These have been listed, after proper filtering, among the robust and reproducible SNPs in other Axiom genotyping arrays [45, 46, 51]. Following the large-scale genotyping with the 70 K array, only 663 and 78 SNPs were classified as NMH and OTV, respectively, and 533 more as *ABvarianceY* (which we could add to the OTV category, as shown in Additional file 12), and they were generally characterized by very low-quality clustering. This was as expected, however, since the 71,363 SNPs of this array were selected amongst robust PHR SNPs. In contrast, higher numbers were found for the 700 K array. While only 29% of the NMH were removed by the stringent filtering and the Mendelian check, the number of discarded OTV was much higher, reaching a proportion of 77%, indicating that genotypic errors are more common in this category. The low polymorphism of the NMH in comparison with the PHR suggests that these SNPs may tag genomic regions that are highly conserved across the species. On the other hand, the low hybridization cluster of the OTV could be due to deletions, non-homology or presence of secondary polymorphism in the probe sequence, and it is expected in samples with a high divergence from the reference genome used to design the SNP markers [68]. The unknown polymorphism of the OTV SNPs was often called “null” allele [27, 69, 70]. The genotypic calls of some samples for these SNPs can be re-coded as A0 (= AA), AB (=AB), B0 (=BB) and 00 (= NoCall). In our sample set, we observed that often samples from Groups 3 and/or 4, which include the Oriental pears that are more genetically divergent from *P. communis*, fell into the additional OTV cluster, which is homozygous for the “null” allele (00), or into the A0 or B0 clusters (heterozygous for the “null” allele) (Additional file 13). As pointed out by Didion et al. [68], by selecting only SNPs that performed well in the screening panel, we have probably introduced an ascertainment bias against rare alleles of underrepresented species. If we had included correct OTV SNPs, as well as robust PHR, we could have, at least partially, counteracted this ascertainment bias, at the expense of a higher risk of miscall and no-call rates for the final 70 K array. However, our screening panel included 27 of the 33 *Pyrus* species held at the USDA-NCGR, plus several unknown interspecific hybrids, and the number of accessions for each species (and group of species) was chosen according to the final proportions in the germplasm collection (Fig. 5 and Additional file 14). Furthermore, by incorporating 11,597 discriminative SNPs (16% of the 70 K array) (Table 3 and Table 4), we included alleles that are rare within each group of species.

## Conclusion

The Axiom Pear 70 K Genotyping Array, with its demonstrated high efficiency across a widely diverse panel of *Pyrus* species, represents a valuable resource for a multitude of molecular genetic studies in pear. Currently, only a mixed *Malus* and *Pyrus* SNP array that includes ~ 1000 pear SNPs [27] is available to the pear research and breeding community. GBS has been applied with success in pear [18, 31, 32]; however, we have shown that the genotypic data generated with a highly efficient SNP array, such as the one described in this study, are easier to analyze, requiring less bioinformatics capacity, and are more robust and reliable, usually including fewer missing values and erroneous calls. The Axiom Pear 70 K Genotyping Array will be useful to quickly generate high-density genotypic information for new germplasm or future breeding or mapping populations internationally. This SNP array is commercially available to the community through Thermo Fisher Scientific.

The USDA-NCGR is a public resource that offers a valuable opportunity to evaluate the relatively unexplored genetic diversity of *Pyrus*. We are in the process of completing the genotyping of the entire collection. The characterization of this germplasm will provide important information to geneticists and breeders, as well as assisting in the optimization of conservation strategies for *Pyrus*.

## Methods

### Plant material and re-sequencing

The polymorphism discovery panel was composed of 55 pear accessions that were selected from the NCGR in Corvallis, OR and the Appalachian Fruit Research Station (AFRS) in Kearneysville, WV (Additional file 1). DNA was extracted from freeze-dried leaves at University of California (UC), Davis using the EZNA HP Plant DNA Mini Kit (Omega Bio-Tek, Inc., Norcross, USA) and quantity and quality of the DNA were checked with a Qubit® 2.0 Fluorometer and a NanoDrop 1000 Spectrophotometer (Thermo Fisher Scientific, Waltham, USA), respectively. For each sample, paired-end libraries were constructed using the Nextera DNA Library Preparation kit (Illumina Inc., San Diego, CA, USA), which were then sent to the Institute for Genomic Medicine at UC San Diego for whole-genome sequencing. Sequencing of the 55 libraries was performed on eight lanes of Illumina® HiSeq2500 in high output mode with v4 chemistry and 2 × 100 bps.

### Read mapping and variant calling

Raw reads of the 55 different pear accessions were quality-evaluated with FastQC v0.11.5 [71], and then quality and adapter-trimmed (using a threshold of 20 for both ends) with cutadapt v1.3 [72]. The sequencing depth was calculated for each sample by dividing the total number of trimmed bp by 516 Mbp (the average of the estimated *P. communis* genome sizes according to Arumuganathan and Earle [73]). Samples were divided into six groups, according to their known common origin [60]: i) Group Communis, including all *P. communis* cultivars and the *P. communis* subsp. *caucasica* and *P. communis* subsp. *pyraster* accessions; ii) Group 1, including species that are considered wild relatives of *P. communis*; iii) Group 2, including Middle East/Central Asia arid-adapted species; iv) Group 3, including East Asian “pea” pear species; v) Group 4, including East Asian large-fruited cultivars and wild relatives; and vi) Group Hybrids, including all supposed interspecific hybrids (Additional file 1). The objective was the grouping together of accessions with expected similar genomes prior to the application of ad hoc parameters for both read mapping and SNP calling. The trimmed reads were mapped the to the ‘Bartlett’ v1.0 reference genome [54] using BFAST v0.7.0 [74] and those with multiple alignments and high mismatches (4% mismatches for the Group Communis and 7% mismatches for the other five groups) were eliminated. Afterwards, the aligned reads of all accessions within each group were merged and treated as unique samples, PCR duplicates were removed with Picard, and polymorphisms were mined against the reference genome using SAMtools v1.3.2 (mpileup) and bcftools v1.2 (calls made with the multiallelic-caller method) [75].

### SNP filtering

Variants were subjected to two filters, the Quality and the Affymetrix filters, to remove artefacts and guarantee a final set of high-quality SNPs (Fig. 1). The Quality filter retained variants with: i) Phred-scaled quality score (QUAL) 19; ii) more than 75% of high quality reads; iii) raw read depth (DP) average sequence depth multiplied by the number of accessions; and iv) DP lower than average DP plus three standard deviations (SDs), where parameters at points iii and iv were calculated independently for each of the six VCF files. Afterwards, all the detected variants were combined into a unique VCF file, also adding 1139 pear SNPs from the ﻿apple and pear Infinium® II 9 K SNP array [27] and 9151 GBS SNPs developed at PFR [32]. Then, all duplicates were removed, and this list of unique variants was submitted to Affymetrix for quality scoring, along with 4741 GBS SNPs that had been mapped to the *P.* x *bretschneideri* ‘Dangshansuli’ genome [55]. Based on the Affymetrix scoring, the following additional filters were applied to the newly discovered variants, keeping only i) SNPs with just two alleles and no INDELs; ii) SNPs for which at least one probe was recommended by Affymetrix (pConvert 0.6); iii) SNPs with no additional polymorphisms within 20 bp up/downstream; iv) SNPs with a maximum DP of 605 (= sum of max DPs used for each group in the Quality filter); and v) SNPs with no more than one missing genotype (Fig. 1). SNPs from the validated set (Illumina and GBS) were kept if i) they had at least one probe either recommended or neutral; ii) they were different from A/T and C/G; and iii) they had been uniquely mapped to a genetic location, according to the maps developed by Montanari et al. [27] and Li et al. [32] (Fig. 1).

### SNP selection for the first “draft” genotyping array

The software SnpEff v4.0 [52] and the gene predictions for ‘Bartlett’ v1.0 (available at https://www.rosaceae.org/species/pyrus/pyrus_communis/genome_v1.0) were used to estimate the impact of SNPs on proteins. In the SnpEff output, SNPs were divided into four categories: i) HIGH impact, ii) MODERATE impact, iii) LOW impact, and iv) MODIFIER. All SNPs with HIGH or MODERATE impact were kept; all the LOW impact SNPs were also kept, except for those with all homozygous in silico genotypes (Fig. 1). From the MODIFIER SNPs, those with all homozygous genotypes, with missing genotypes, the A/T and C/G SNPs and the intergenic SNPs (as from SnpEff) were removed. All these SNPs were submitted to Affymetrix as high priority for the array design, along with the validated SNPs. Finally, the remaining intergenic SNPs were given low priority for the array design, and a sorted list was submitted to Affymetrix. A FP strategy was used to prioritize the intergenic SNPs. One FP was placed every 20 Kbp on the reference genome, or in the middle of scaffolds shorter than 20 Kbp, and then a window of 10 Kbp from each side of the FP was considered. SNPs with the higher number of heterozygous genotypes for each FP window were prioritized; these were followed by the second set of SNPs with the higher number of heterozygous genotypes from each FP, and so on. At Affymetrix, a custom 700 K genotyping array was built according to the Axiom myDesign™ protocol, tiling first the SNPs from the high priority file, and then the SNPs from the low priority file starting from the top of the list and moving down, until completion.

### Screening panel

A number of 284 diverse pear accessions was selected as the screening panel to be genotyped with the Axiom Pear 700 K Genotyping Array (Additional file 3). These included: 268 accessions representative of the entire diversity held at the NCGR collection; 11 founders of the AFRS pear breeding program; three founders of the French breeding program at The Institut National de la Recherche Agronomique (INRA) in Angers; a cultivar important to PFR in New Zealand; and a DH of ‘Bartlett’, generated and currently grown at INRA in Angers [76] and being used for the development of a new high-quality *P. communis* genome assembly (‘Bartlett’ v2.0). A total of three technical replicates of ‘Bartlett’ (CPYR 38.001), one per plate, two of *P. pyrifolia* ‘Dan Bae’ (CPYR 2623.001) and two of the DH were also included. DNA was extracted and evaluated as described above and sent to Affymetrix to be genotyped with the Axiom Pear 700 K Genotyping Array.

### Selection of highly-informative SNPs for the final genotyping array

The genotypic data from the screening panel were analyzed using particularly stringent metrics, to identify the most robust SNPs (Fig. 2). The default Affymetrix parameters were used for initial QC of the samples: Dish QC ≥ 0.82 and QC CR ≥ 97% (for detail see Affymetrix Axiom Genotyping Solution – Data Analysis Guide, http://www.bea.ki.se/documents/axiom_genotyping_solution_analysis_guide.pdf). Samples that did not pass these thresholds were excluded from the analysis. Genotypic calls and SNP QC performed at Affymetrix were then modified by applying more stringent thresholds. First, the function *Ps_CallAdjust* in the R package “SNPolisher” v1.5.2 was used to decrease the confidence threshold from the default 0.15 to 0.01 (samples with a confidence score 0.01 were assigned no call), and then the functions *Ps_Classification* and *Ps_Classification_Supplemental* were run to divide the SNPs into 13 different categories based on their QC metrics (Table 2). The SNPs for which the technical replicated samples (three ‘Bartlett’, two ‘Dan Bae’ and two DH) exhibited different genotypes and the SNPs that were called heterozygous in the DH were removed. Subsequently, the SNPs were checked for Mendelian errors, by examining the segregation in 22 trios (Additional file 4) and using the function *trio.check* in the R package “trio” v3.8.0 [77] in “Bioconductor”. All trios with a Mendelian error rate > 10% were removed, *trio.check* was re-run and finally the SNPs with an error rate 5% across the true trios were eliminated. Finally, more stringent metric thresholds were applied, assigning SNPs to the category PHR if they had CR ≥ 98%; Fisher’s Linear Discriminant (FLD) 6.6; Homozygous FLD (HomFLD) 14.3, Heterozygous Strength Offset (HetSO) 0; n_AB ≠ 0; all other metric thresholds were left as default. These SNPs were defined as robust PHR.

For the final selection of the markers to include in the Axiom Pear 70 K Genotyping Array, all the A/T and C/G SNPs were removed, since those require double probes on the array (Fig. 2). Then, the remaining SNPs (with their 35 bp-flanking sequences) were aligned to the current (May 2017) assembly of ‘Bartlett’ v2.0 (G. Linsmith, unpublished) using BLAST [78]. Specifically, we used the BLAST® command line application [79] v2.2.29 to first build a database of the genome (makeblastdb -dbtype nucl), and then run blastn (blastn -task blastn-short). With a custom R script, we subsequently filtered the alignments, by keeping only the queries that had unique alignments with identity 93%, alignment length/probe length 92 and 108%, and e-value 1 *e*^−20^. Afterwards, FPs were placed every 100 Kbp on the new assembly and SNPs in high LD (*r*^2^> 0.85) within each 100 Kbp FP window were removed. Afterwards, the SNPs inside each FP window were prioritized according to their SnpEff prediction (HIGH, MODERATE, LOW and MODIFIER) and to their degree of polymorphism. For this last point, values of MAF across the entire screening panel and within each group of species were used to identify rare (MAF 0.2 and MAF 0.8) and common SNPs (0.2 ≤ MAF ≤ 0.8), and to divide the SNPs into classes of polymorphism (Table 3). In the FP-based selection SNPs that were polymorphic across the entire screening panel and within the Group Communis were prioritized, along with those that could discriminate between two or more groups (i.e. SNPs with different minor alleles in groups with MAF 0.2 and MAF 0.8). In addition, SNPs from the Infinium® II chip that had been associated with important agronomic traits [7, 35] were included. This prioritized list of SNPs was submitted to Affymetrix for the construction of the final Axiom Pear 70 K Genotyping Array.

SNPs that were successfully tiled to the Axiom Pear 70 K Genotyping Array were aligned to the *P. communis* ‘Bartlett’ v1.1 [32, 54] and *P.* x *bretschneideri* ‘Dangshansuli’ [55] genomes. Alignments and filtering were performed as described above, with the exception of the identity threshold, which for *P.* x *bretschneideri* was reduced to 90%. Each chromosome for both genomes was then divided into bins of 500 Kbp, and the number of SNPs in each bin counted and plotted over the chromosome length.

### Principal component analysis

The population structure of the screening panel was evaluated using both the robust PHR SNPs and the subset of highly-informative SNPs that were to be included in the Axiom Pear 70 K Genotyping Array. First, the Concordance Check in the Axiom Analysis Suite v2.0 software was run to flag duplicated samples (with values of concordance 97%), and only one was kept for subsequent analysis. Putative sampling errors were verified by SSR fingerprinting the original trees from the NCGR collection (Additional file 6), using a standard set of 12 microsatellites developed as a result of a European Cooperative Programme for Plant Genetic Resources (ECP/GR) workshop on *Pyrus*, *Malus* and *Prunus* [42, 80]. Then, PCA was performed using the R package “SNPRelate” [81] and the results for the entire list of robust PHR and for the selected SNPs were compared.

### Validation of the 70 K array through large-scale genotyping

A total of 1416 samples were genotyped with the Axiom Pear 70 K Genotyping Array. This genotyping panel included: 1358 *Pyrus* accessions from the NCGR (with 30 duplicates); two pear rootstocks collected from a commercial orchard in Ukiah, CA; three and seven founders of the AFRS and the INRA-Angers pear breeding programs, respectively; 12 samples from Fondazione Edmund Mach (FEM), including nine historical Italian varieties and three different clones of ‘Bartlett’; one haploid ‘Bartlett’ and two different samples of the DH of ‘Bartlett’ from the INRA of Angers (Additional file 8). Several biological and technical replicates of our reference ‘Bartlett’ (CPYR 38.001) were included as controls, one in each of the 20 plates, as well as biological replicates of some important samples. The accuracy of the default genotypic calls was evaluated by visually examining the cluster plots of 1000 PHR SNPs. Then, the genotypes of all samples were merged with those from the screening panel, filtered for the SNPs included in the 70 K array that were classified as PHR, duplicated samples were eliminated and another PCA was performed as described above.

### Linkage map construction

An interspecific segregating population (P16.009) was developed by crossing two advanced selections from the PFR cultivar breeding program, and raising 57 seedlings. DNA was extracted from the two parents and the offspring using the Qiagen DNeasy™ Plant Mini Kit (Qiagen Inc., Valencia, CA, USA). Samples were genotyped with the Axiom Pear 70 K Genotyping Array. Genetic map construction was carried out using JoinMap 5.0® [82] (www.kyazma.nl/index.php/JoinMap/) with the SNP markers segregating as backcross type for each parent, following the double pseudo-testcross strategy [83], and removing identical markers. The genetic distance within the group was calculated using the Kosambi function, and the regression mapping algorithm was used for map calculation with default parameters. The Linkage Group (LG) numbers were assigned basing on the position of the SNP markers on currently anchored scaffolds of the genome ‘Bartlett’ v1.0, thus consistently with previously published pear genetic maps.

### Calculation of error rates for SNP array and GBS data

The Axiom Pear 70 K Genotyping Array was employed to genotype the two parents and 16 offspring of the F1 interspecific population P493 from the PFR breeding program. GBS-based genotypic data generated with the restriction enzyme reduced representation method [56] were also available for these individuals. The accuracy of the SNP array and the GBS method was tested by counting how many unexpected genotypes were observed in the 16 progenies (for example, for a AG x AA SNP, the number of GG genotypes were recorded), and then by computing the percentage of erroneous data points over all genotypic data points (Additional file 11).

### Evaluation of additional classes of SNPs

The cluster plots for a subset of SNPs from the categories NMH, *OffTargetVariance*, *AAvarianceX*, *AAvarianceY*, *ABvarianceX*, *ABvarianceY*, *BBvarianceX* and *BBvarianceY* (Table 2), both from the 700 K and the 70 K arrays, were visually evaluated to ascertain if the particular classification was due to low quality probes or to genotyping errors. When a large number of the observed SNPs showed a clear cluster separation, the entire class was subjected to a stringent filter, using the metric thresholds applied earlier to the PHR (NMH: CR ≥ 98%; FLD 6.6; HetSO 0; OTV: CR ≥ 98%; FLD 6.6; HomFLD 14.3 or NA), and their Mendelian error rate in the trios was checked. SNPs that passed these two filtering steps were considered robust and reproducible.

## Additional files

Additional file 1: Polymorphism discovery panel. The 55 *Pyrus* accessions re-sequenced for variant discovery. For each sample the table shows the accession’s Plant Introduction (PI) number, the inventory lot identifier, the assigned taxon and common plant name, the group to which the species belongs (as in Challice and Westwood [60]), the source of the sample, the total number of trimmed read pairs (original 100 bp paired-end reads were adapter and quality-trimmed), and the average coverage. (XLSX 16 kb)

Additional file 2: Explanation of the SnpEff categories for all annotated variants. The number of variants for each type of predicted impact on the gene and the explanation of the effects are shown, as reported in the SnpEff manual (http://snpeff.sourceforge.net/SnpEff_manual.html). (XLSX 10 kb)

Additional file 3: Screening panel. The 288 *Pyrus* accessions screened with the Axiom™ 700 K Pear Genotyping Array. For each sample, the Table shows the accession’s Plant Introduction (PI) number, the inventory lot identifier, the assigned taxon and common plant name, the origin, the group to which the species belongs (as in Challice and Westwood [60]), the source of the sample, if it failed or passed quality check and the reason for failure. (XLSX 28 kb)

Additional file 4: List of trios used for Mendelian check. A total of 22 trios (two parents, P01 and P02, and one offspring, Off) were included in the screening panel and were used for the Mendelian test on the *PolyHighResolution* SNPs of the Axiom™ 700 K Pear Genotyping Array. (XLSX 12 kb)

Additional file 5: Distribution of the SNPs of the Axiom™ 70 K Pear Genotyping Array on the pear genomes. The number of SNPs in 500 Kbp bins was plotted over each chromosome length for the *P. communis* ‘Bartlett’ v1.1 [32, 54] (on top) and the *P.* x *bretschneideri* ‘Dangshansuli’ [55] (on the bottom) genomes. The red dashed lines show the average number of SNPs per bin for each chromosome. The table in the center reports the total number of SNPs uniquely aligned to each chromosome (chr) of the two genomes, as well as those aligned to unanchored scaffolds (0). (PDF 174 kb)

Additional file 6: Concordance Check results for the samples of the screening panel. Pairwise concordance values 97% are reported, for a total of 19 groups of duplicated samples (three ‘Bartlett’ pairwise concordances count as one group). Putative sampling errors were verified by SSR analysis. (XLSX 11 kb)

Additional file 7: Principal Component Analysis (PCA) plots of the PC pairs for the first four PCs. (A) PCA performed with all robust *PolyHighResolution* (PHR) SNPs on the screening panel. (B) PCA performed with the SNPs tiled on the Axiom Pear 70 K Genotyping Array on the screening panel. (C) PCA performed with the PHR SNPs of the Axiom Pear 70 K Genotyping Array on all genotyped accessions, including both the screening and the genotyping panel. A different color is used for each *Pyrus s*pecies. Group Communis = *P. communis*; Group 1 = *P. communis* wild relatives; Group 2 = Middle East/Central Asia arid-adapted species; Group 3 = East Asian “pea” pears; Group 4 = East Asian large-fruited cultivars and wild relatives; Group Hybrids = interspecific hybrids. (PNG 1146 kb)

Additional file 8: Genotyping panel. The 1416 *Pyrus* accessions genotyped with the Axiom™ 70 K Pear Genotyping Array. For each sample the table shows the accession’s Plant Introduction (PI) number, the inventory lot identifier, the assigned taxon and common plant name, the origin, the group to which the species belongs (as in Challice and Westwood [60]), the source of the sample, if it failed or passed and the reason for failure, and the ploidy. (XLSX 94 kb)

Additional file 9: Parental genetic maps of the F1 population P16.009 constructed with the Axiom™ Pear 70 K Genotyping Array. Genetic map of the female parent is on page 1, that of the male parent on page 2. (PDF 157 kb)

Additional file 10: Statistics about the parental genetic maps of the F1 population P16.009. The number of markers, the length in cM, the average distance between markers (in cM) and the length of the largest gap (in cM) are reported for each Linkage Group (LG) and for the two maps. (XLSX 11 kb)

Additional file 11: Comparison of SNP array and GBS data error rate. The total number of Axiom™ 70 K Pear Genotyping Array and GBS-based SNP markers segregating in a backcross manner in the P493 population are reported. The total numbers of data points and erroneous data points were counted in 16 offspring for each segregation type. The total numbers of heterozygous × homozygous (Het × Homo) and homozygous × heterozygous (Homo × Het) segregation types were calculated for both Axiom and GBS SNPs. (XLSX 11 kb)

Additional file 12: Cluster plots of an *ABvarianceY* and an *OffTargetVariant* SNP of the Axiom™ Pear 700 K Genotyping Array. The SNP on the left-hand side was classified as *ABvarianceY*, the SNP on the right-hand side as *OffTargetVariant*. Samples are from the screening panel. (PNG 629 kb)

Additional file 13: Cluster plots of two OTV SNPs of the Axiom™ Pear 700 K Genotyping Array with samples colored by species. Both SNPs were classified as OTV (*ABvarianceY* or *OffTargetVariant*) and were processed with the *OTV_Caller* function in “SNPolisher”. Samples are from the screening panel and different colors are used for each *Pyrus* species. Species in green color gradients belong to Group Communis (*P. communis*) or Group 1 (*P. communis* wild relatives); species in red color gradients belong to Group 2 (Middle East/Central Asia arid-adapted species); species in purple/pink color gradients belong to Group 3 (East Asian “pea” pears); species in blue color gradients belong to Group 4 (East Asian large-fruited cultivars and wild relatives); species in yellow color gradients belong to Group Hybrids (interspecific hybrids). (PNG 1213 kb)

Additional file 14: Number of samples for each species in the screening panel and in the USDA-NCGR collection. The number of samples for each *Pyrus* species are reported for the screening panel and for the entire USDA National Clonal Germplasm Collection of Corvallis, OR. (XLSX 11 kb)

## Abbreviations

AFRS: Appalachian Fruit Research Station
BLAST: Basic local alignment search tool
CR: Call rate
DH: Double haploid
DP: Read depth
ECP/GR: European Cooperative Programme for Plant Genetic Resources
FEM: Fondazione Edmund Mach
FLD: Fisher’s Linear Discriminant
FP: Focal point
GBS: Genotyping-by-sequencing
GRIN: Germplasm Resources Information Newtork
GS: Genomic selection
GWAS: Genome-wide association studies
HetSO: Heterozygous Strength Offset
HomFLD: Homozygous FLD
INDELs: Insertions/deletions
INRA: Institut National de la Recherche Agronomique
LD: Linkage disequilibrium
LG: Linkage group
M: Million
MAF: Minor allele frequency
MAS: Marker-assisted selection
NMH: *NoMinorHomozygous*
OTV: *Off-TargetVariants*
PCA: Principal component analysis
PFR: Plant & Food Research Limited
PHR: *PolyHighResolution*
PI: Plant Introduction
QC: Quality control
QTL: Quantitative trait locus
QUAL: Phred-scaled quality score
SD: Standard deviation
SNP: Single nucleotide polymorphism
UC: University of California
USDA-NCGR: United States Department of Agriculture – National Clonal Germplasm Repository
VCF: Variant call format
