# Supplementary material for: Development of a highly efficient Axiom™ 70 K SNP array for Pyrus and evaluation for high-density mapping and germplasm characterization
Source: BMC Genomics. 2019 May 2;20:331. doi: 10.1186/s12864-019-5712-3 (PMC6498479; doi:10.1186/s12864-019-5712-3)

'Bartlett' v1.1

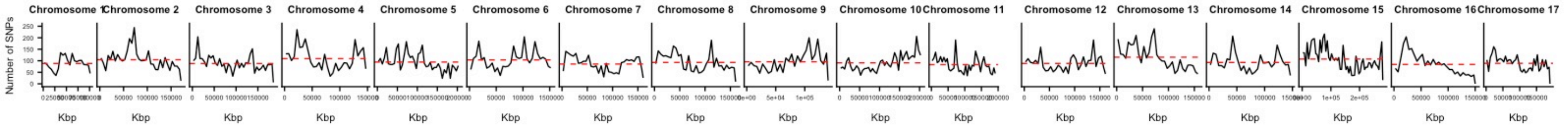

	chr 1	chr 2	chr 3	chr 4	chr 5	chr 6	chr 7	chr 8	chr 9	chr 10	chr 11	chr 12	chr 13	chr 14	chr 15	chr 16	chr 17	0
**'Bartlett' v1.1**	1858	3660	3351	3275	3869	3213	2832	3064	2774	3726	3247	2923	3696	2782	6127	2611	3471	12708
**P. bretschneideri**	1382	2913	3022	1691	3320	2434	1451	1945	2432	2361	2250	2255	2559	2070	5267	1625	2202	2637

P. bretschneideri

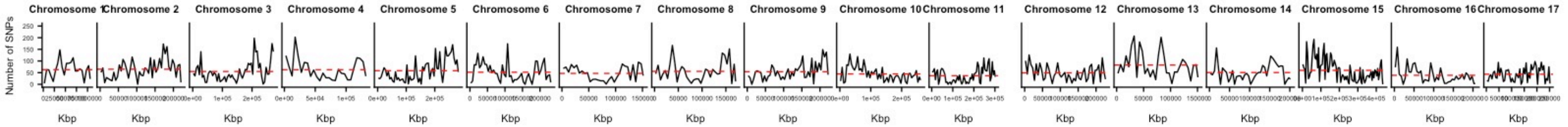

Supplement: Supplementary file 5 — Distribution of the SNPs of the Axiom™ 70 K Pear Genotyping Array on the pear genomes. The number of SNPs in 500 Kbp bins was plotted over each chromosome length for the P. communis ‘Bartlett’ v1.1 [32, 54] (on top) and the P. x bretschneideri ‘Dangshansuli’ [55] (on the bottom) genomes. The red dashed lines show the average number of SNPs per bin for each chromosome. The table in the center reports the total number of SNPs uniquely aligned to each chromosome (chr) of the two genomes, as well as those aligned to unanchored scaffolds (0). (PDF 174 kb) [file 12864_2019_5712_MOESM5_ESM.pdf]
