# Supplementary figures and images for: Development of a highly efficient Axiom™ 70 K SNP array for Pyrus and evaluation for high-density mapping and germplasm characterization
Source: BMC Genomics. 2019 May 2;20:331. doi: 10.1186/s12864-019-5712-3 (PMC6498479; doi:10.1186/s12864-019-5712-3)

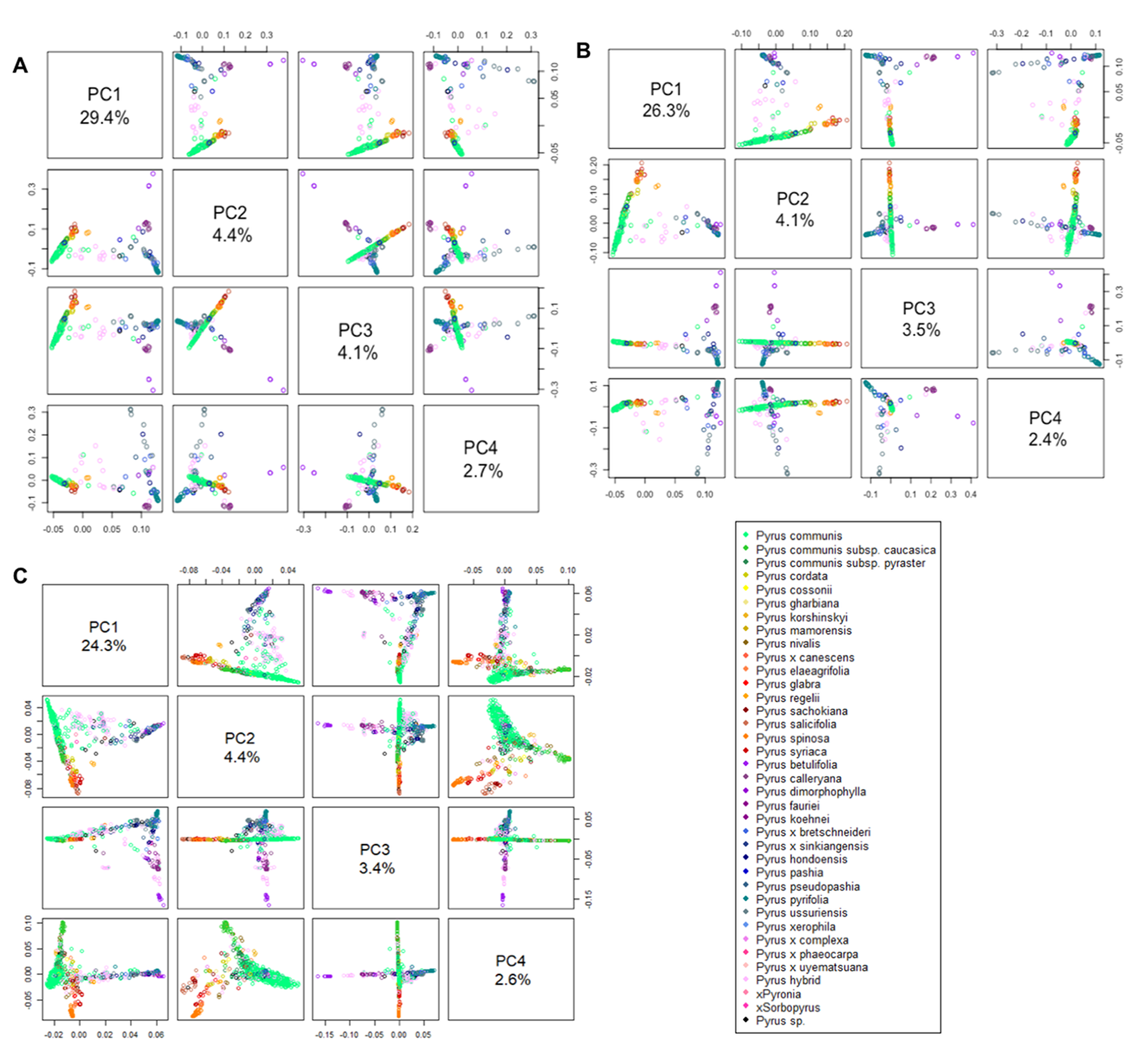

Supplement: Supplementary file 7 — Principal Component Analysis (PCA) plots of the PC pairs for the first four PCs. (A) PCA performed with all robust PolyHighResolution (PHR) SNPs on the screening panel. (B) PCA performed with the SNPs tiled on the Axiom Pear 70 K Genotyping Array on the screening panel. (C) PCA performed with the PHR SNPs of the Axiom Pear 70 K Genotyping Array on all genotyped accessions, including both the screening and the genotyping panel. A different color is used for each Pyrus species. Group Communis = P. communis; Group 1 = P. communis wild relatives; Group 2 = Middle East/Central Asia arid-adapted species; Group 3 = East Asian “pea” pears; Group 4 = East Asian large-fruited cultivars and wild relatives; Group Hybrids = interspecific hybrids. (PNG 1146 kb) [file 12864_2019_5712_MOESM7_ESM.png]

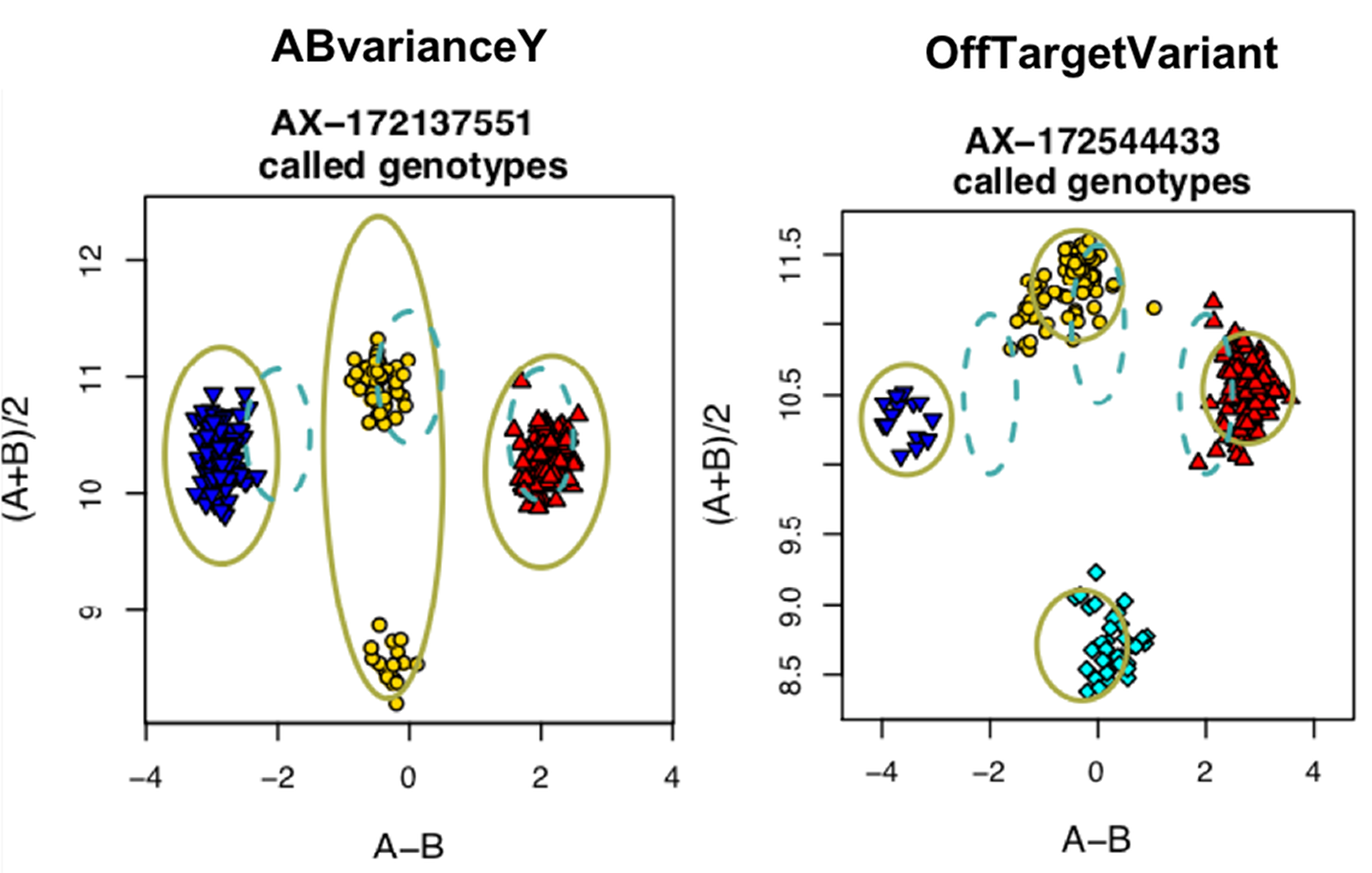

Supplement: Supplementary file 12 — Cluster plots of an ABvarianceY and an OffTargetVariant SNP of the Axiom™ Pear 700 K Genotyping Array. The SNP on the left-hand side was classified as ABvarianceY, the SNP on the right-hand side as OffTargetVariant. Samples are from the screening panel. (PNG 629 kb) [file 12864_2019_5712_MOESM12_ESM.png]

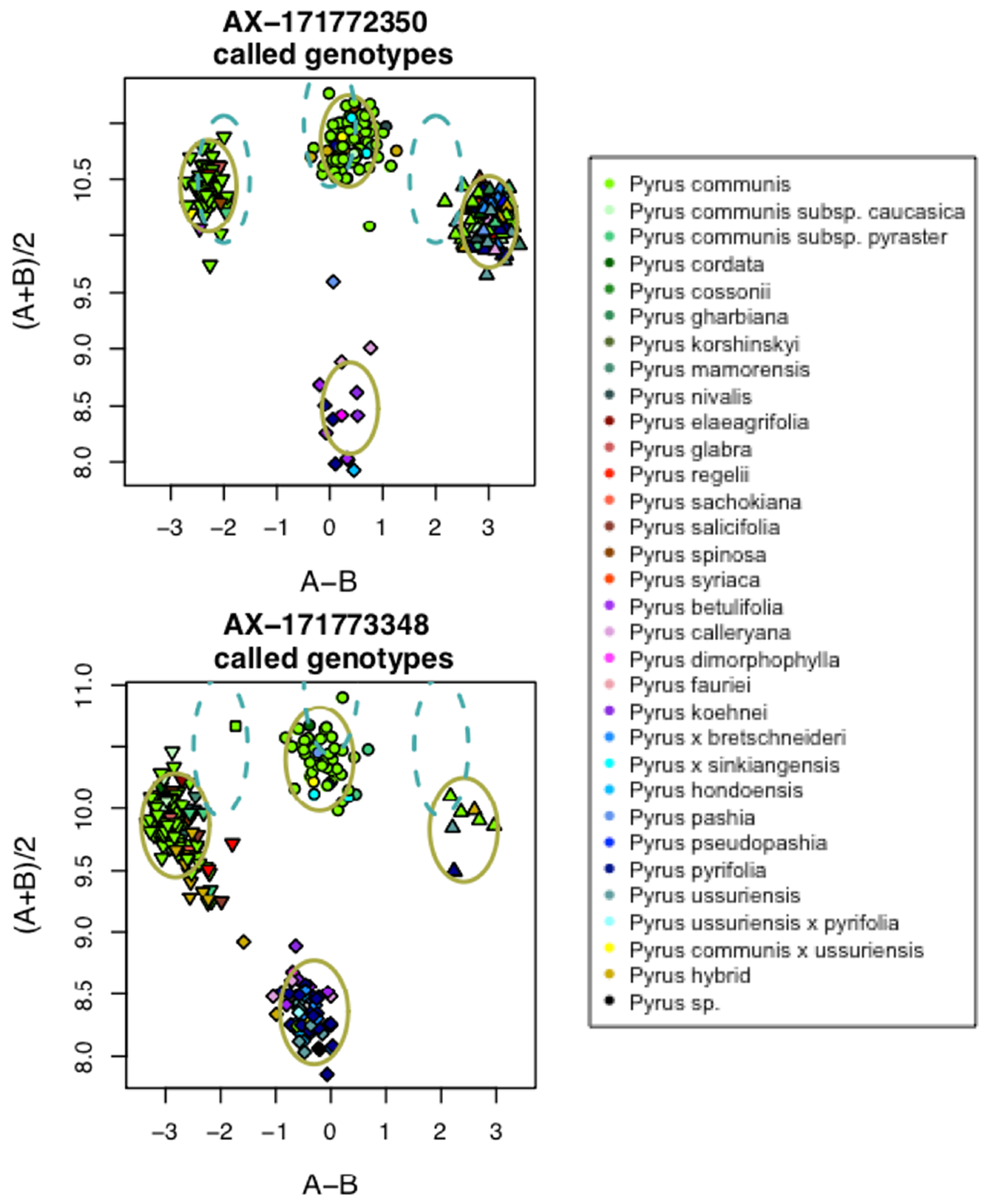

Supplement: Supplementary file 13 — Cluster plots of two OTV SNPs of the Axiom™ Pear 700 K Genotyping Array with samples colored by species. Both SNPs were classified as OTV (ABvarianceY or OffTargetVariant) and were processed with the OTV_Caller function in “SNPolisher”. Samples are from the screening panel and different colors are used for each Pyrus species. Species in green color gradients belong to Group Communis (P. communis) or Group 1 (P. communis wild relatives); species in red color gradients belong to Group 2 (Middle East/Central Asia arid-adapted species); species in purple/pink color gradients belong to Group 3 (East Asian “pea” pears); species in blue color gradients belong to Group 4 (East Asian large-fruited cultivars and wild relatives); species in yellow color gradients belong to Group Hybrids (interspecific hybrids). (PNG 1213 kb) [file 12864_2019_5712_MOESM13_ESM.png]
